# The Medical Schools

**Published:** 1900-09-08

**Authors:** 


### The University of St. Andrews.

### The Medical Schools.

The following is a complete list of all the medical schools in the United Kingdom, giving, as far as the English schools are concerned, the number of beds in the hospital to which each school is attached; the amount of the composition fee (the fee charged for admission to all the lectures, hospital practice, &c., required for the diplomas of the Conjoint Examining Board); some particulars as to the scholarships and prizes which are offered; such other matters as may be of interest, and finally the postal address. These cover most of the points on which those in search of a school require some general information for their guidance. On application being made to any of these medical schools, full details, usually in the form of a printed syllabus of the course of study, fees, prizes, &c., will be forwarded. The official to whom application should be made is at some schools called the Dean, at others the Warden, at others simply the Secretary, but the most usual form of address is "The Dean of th Medical School."

The information given below is intended principally for those commencing their medical curriculum. It must be understood, however, that for those who have already entered, say at Cambridge or Durham, and who wish to do the second half of their course in London, one of the most important matters as to which inquiry should be made is as to how far a man so changing his school in the middle of his curriculum would be eligible for the higher hospital appointments, such as house surgeon, house physician, &c.

### LONDON MEDICAL SCHOOLS.

### St. Bartholomew's Hospital Medical School, *West Smithfield, E.C.*

The hospital contains 744 beds, including those at the convalescent branch hospital at Swanley, and is very well equipped in regard to special departments, thus offering a large number of appointments to the students. The following paid appointments may properly be regarded as valuable prizes to the senior students: Ten house physicians, ten house surgeons, a resident midwifery assistant, and an ophthalmic house surgeon, two assistant anæsthetists, and an extern midwifery assistant.

The total value of the scholarships and prizes is nearly £900 per annum. There is a resident college for students within the precincts of the hospital, and a very admirable recreation ground has been acquired at Winchmore Hill, which is easily reached by rail.

The composition fee is 150 guineas.

### Guy's Hospital Medical School, *London Bridge, S.E.*

The hospital contains more than 600 beds, of which 550 are in constant occupation. The special departments are well organised, and not only is there a special ward for the diseases of women, but a few beds are reserved for cases of difficult labour.

A peculiarity of Guy's is what are called the clinical wards—wards set apart for the most urgent and interesting medical cases, which form the subjects of the weekly clinical lectures. A very large number of appointments are open to the students, and although unpaid they are valuable prizes; besides which rooms and commons are provided for all the "indoor" appointments, and a large number of scholarships and prizes are open to the students.

There is a resident college, providing accommodation for 60 resident students, and dining hall, reading-rooms, &c., forming a sort of club for members of the Clubs Union. The athletic ground at Honor Oak Park is easily accessible.

A large number of scholarships and prizes are given every year.

The composition fee is 150 guineas.

### St. Thomas's Hospital Medical School, *Albert Embankment, S.E.*

The hospital contains 572 beds, of which about 512 are in use, and available for clinical teaching. Alterations and improvements have been going on for some time, which make it in many points one of the most perfect, if not the most perfect, of the great general hospitals of London. Among other improvements may be mentioned the two clinical theatres in the out-patient department, which enable the vast field of the out-patients to be made much more use of for clinical instruction than can be the case at some hospitals.

The school buildings are very well equipped. There are many valuable scholarships and prizes, and all the hospital appointments are open to the students without charge. Several of the resident appointments are well paid.

There is no resident college, but the position of the hospital in regard to railways and tramways makes it very easy for students to live in the suburbs during their earlier terms; often a great advantage.

The composition fee is £150.

### King's College Medical Faculty, *Strand, W.C.*

King's College Hospital is situated in Portugal Street, Lincoln's Inn, W.C., about five minutes' walk from the colleges. It contains 220 beds.

In the college there are resident chambers for a certain number of students. It may be stated here that King's College is founded on a very wide basis, and that its educational scheme embraces many faculties besides that of medicine. Thus the medical students at King's College, as also those at University College, form but a part of a large body of students working at a large range of subjects. We are afraid that both at King's and University the medical students keep much together, but one cannot but feel that it ought to be possible to gain much and to widen one's view of things very considerably from associating during one's student life with men working at a variety of subjects.

There are many entrance scholarships, and during the curriculum scholarships and prizes of the value of £300 are open to matriculated students.

The College has a large recreation ground at Wormwood Scrubbs.

The composition fee is £135.

### University College Medical Faculty, *Gower Street, W.C.*

The hospital and the college stand opposite to each other in Gower Street, and what we have said in regard to King's College as to the possible advantages of the medical students being but a part of a large mass of students working at many other subjects applies equally to University.

The hospital contains 185 beds, but the patients are just now being transferred to the fine new hospital, a part of which is ready for occupation, and students now entering will have the advantage of working in a new and very fine building quite up-to-date in every direction.

There are some good entrance scholarships, and other exhibitions, prizes to the annual amount of about £500 being given in the Faculty of Medicine.

The composition fee is 135 guineas.

### Charing Cross Hospital Medical School, *Charing Cross, W.C.*

The hospital contains 180 beds, and is now being enlarged, and students are admitted, for their eye work, to the Royal Westminster Ophthalmic Hospital, which is adjacent, and contains 30 beds.

For a hospital of its size there are a considerable number of house appointments. From its peculiar position, in one of the busiest centres of London, the Charing Cross Hospital is kept constantly in full swing, and offers a great field in certain departments.

There are several entrance scholarships, one of which is reserved for sons of medical men, and two to students of a certain standing at Oxford or Cambridge. An Epsom Scholarship, giving a free education, is each winter session placed at the disposal of the Royal Benevolent College, at Epsom, to be competed for by foundation scholars who have passed the Preliminary Scientific M.B. Examination of the University of London.

The composition fee is 110 guineas.

### St. George's Hospital Medical School, *Hyde Park Corner, S.W.*

This hospital stands in a commanding position at Hyde Park Corner; and in addition to serving, as it is popularly supposed to do, as a "servants' hospital" to the two fashionable districts, Mayfair and Belgravia, between which it is planted, is the principal hospital for those districts still further to the west which are becoming rapidly covered with houses of a more or less working-class character.

The hospital contains 351 beds.

There are the usual house appointments, and a considerable number to which salaries are attached are open to the senior students. There are also several entrance and other scholarships.

One of the entrance scholarships of the value of £150 is reserved for the sons of medical men; two, of £85, for students of a certain standing from Oxford or Cambridge; and another similar one for students from the Provincial University Colleges.

The composition fee is £150.

London Hospital Medical School,
*Mile End, E.*

The hospital, which is the largest general hospital in the kingdom, contains nearly 800 beds. Although in the East-end, and in the midst of a vast labouring population, with docks and factories and workshops on every side, the hospital itself, with its attached school, stands in a comparatively open and pleasant position. By its immediate propinquity to railways also, many pleasant suburbs are rendered available as residential quarters for the students during the earlier part of their curriculum.

Naturally, with such an enormous number of patients, the hospital offers not only a great field for the study of disease, but, from the number of appointments which are open to students, gives many opportunities for the personal and responsible care of patients, under supervision, which is perhaps the best method of obtaining a practical knowledge of the art as well as the science of medicine. A fair number of these appointments carry salaries with them.

There are several good entrance scholarships, and the list of prizes and scholarships to be obtained later is considerable. The real prizes at the London, however, are the appointments and the opportunities which they offer.

The school buildings, which are within the hospital grounds, have been considerably enlarged. The new departments for the teaching of bacteriology, public health, chemistry, and biology are now completed and in full use, and during the last year new students' rooms have been added, and a garden and fives court for the use of the members of the Clubs Union have been opened. There is also a Clubs Union athletic ground, which is within easy reach of the hospital.

The composition fee is 120 guineas, a reduction of 15 guineas being made to sons of medical men.

St. Mary's Hospital Medical School,

*Paddington, W.*

The hospital at present contains 281 beds, a number, however, which will be raised to 381 when the Clarence wing is completed. A large new out-patient department was opened a couple of years ago, which offers to the students great advantages in the way of clinical observation among the large number of out-patients constantly attending the hospital. The situation of the hospital, in the midst of a very pleasant residential quarter, offers great advantages to students who wish to live near to their work, while the railway, which is only just across the way, renders access to the country and the river peculiarly easy. All the clinical appointments are free, and the residents, who are chosen by competitive examination, receive board and residence in the hospital. Large alterations and improvements have lately been made in the school buildings, which now offer every advantage for advanced teaching.

There are several good entrance scholarships.

The composition fee is £140 if paid in one sum, and includes membership of the amalgamated clubs. Special arrangements are made for those who have already completed their examinations in anatomy and physiology.

Middlesex Hospital Medical School,
*Berners Street, W.*

The hospital contains 320 beds. A new wing, containing 40 beds, and provided with special research laboratories, is devoted to patients suffering from cancer, the study of which disease has long been made a special feature at this hospital. There are a considerable number of resident appointments, 18 such being awarded annually by open competition among the students of the hospital, and all the officers so elected reside and board in the Residential College free of charge. This residential college is attached to the hospital and overlooks the hospital grounds. It accommodates about 30 students.

There are two open entrance scholarships, as well as one open to students of a certain standing at the universities of Oxford or Cambridge, and one to pupils of the Royal Medical College, Epsom.

The composition fee is 135 guineas.

Westminster Hospital Medical School,
*Caxton Street, S.W.*

The Westminster Hospital is situated opposite Westminster Abbey, the medical school being in Caxton Street. The hospital contains upwards of 200 beds. A medical and a surgical registrar are appointed annually, each with a salary of £40, and the resident officers are provided with rooms and commons. For its size this school is peculiarly well endowed with scholarships, among which is one giving a free medical education to pupils of the Epsom College.

The composition fee is 110 guineas.

London (Royal Free Hospital) School of Medicine for Women,
*30, Handel Street, Brunswick Square, W.*

The London School of Medicine for Women is carried on in connection with the Royal Free Hospital.

This hospital contains 170 beds, all of which are available for clinical instruction, and it is provided with separate departments for the various specialties. Arrangements are made by which students attend the practice of one of the Fever Hospitals of the Metropolitan Asylums Board, and also the practice in mental diseases at one of the institutions under the Asylums Committee of the London County Council.

Several entrance and other scholarships and prizes are open to the students, in addition to which various Missionary Societies offer scholarships or other assistance to ladies who will undertake, after obtaining their qualification, to go out to India as medical missionaries. The course of study provides for all the examinations for qualification open to women.

The school buildings have lately been entirely rebuilt and enlarged, and already contain three large lecture theatres and all necessary laboratories. The third wing, which will be opened in October, will contain common room, library, museums, and sets of chambers available for students.

The composition fee is £125.

### Cooke's Medical School.

Although not a complete school, the school of anatomy founded by the late Mr. Cooke and still carried on by his successors must be mentioned among the medical schools of London, furnishing as it does opportunities to many students for taking out special classes, or for doing the supplementary work in anatomy and physiology required of those who have been rejected at the examinations on these subjects by the conjoint board, &c. The school is also recognised for the special dissection required for the Fellowship of the Royal College of Surgeons, and for the operative surgery required on promotion in the Royal Army Medical Corps.

In connection with this school there is a scholarship, the Bland-Sutton Presentation, which confers the privilege of free education during the first two years of the curriculum.

### PROVINCIAL MEDICAL SCHOOLS.

In all the provincial medical schools the curriculum is so arranged as to give to the students precisely the same opportunities of obtaining degrees and diplomas as are open to those studying in London. They stand, however, on a somewhat different footing from most of the metropolitan schools, more especially in regard to their relation to the hospitals. In fact, but for the great clinical opportunities offered by London, and the attraction which London has always exercised upon medical aspirants, by dint of which much of the best of the talent in the profession is to be found in the metropolis, where it is available for teaching purposes, the position occupied by many of the provincial schools, forming as they do an integral part of great colleges and Universities, would seem higher than that of many of the London schools, which are mere outgrowths of individual hospitals. Take, for example, the Cambridge school, which is part and parcel of the Cambridge University; or the College of Medicine at Newcastle-on-Tyne, which, with its associated faculties of science, &c., is a part of the University of Durham. In Manchester, Liverpool, and Leeds, again, we find great colleges in which advanced teaching in almost every department of knowledge is being carried on, the medical being one among many faculties in colleges, each of which is a great seat of learning. This can only be said of two, and, unfortunately, those two are not the greatest, of the London schools. The glamour of great names, the undoubted advantage of the enormous field for clinical observation offered by the London hospitals, the fact that everything strange and out of the way does in the end come to London, and the very obvious benefit which the student derives from the amount, we will not say of idle talent, but of talent "on its promotion," which is available for teaching purposes at all the London schools, must always make London pre-eminent as a medical school, notwithstanding the purely private position of most of its teaching institutions. But no one need nowadays be ashamed of being a provincial student. The public position occupied by the provincial schools, the admirable hospitals in which the medical instruction is given, and the advanced position occupied by the leading provincial teachers and practitioners, offer to the student everything that is required for obtaining a thoroughly sound professional education, while many of the provincial schools give him far greater opportunities than he has in London of obtaining that public recognition of his work which is expressed by a University degree. It is to be noted that the provincial medical colleges, not being mere appendages of the hospitals, the fees for hospital practice mentioned in the following list are in many cases given separately from those for the college course.

### Cambridge University Medical School.

It is, we believe, possible to go through the whole of the medical curriculum at Cambridge, but it is chiefly as a great science school in which the first half of the curriculum may be advantageously passed that Cambridge takes its high position among seats of medical learning.

The advantages of studying at Cambridge are very great, and for those who can afford it there can be very little doubt that there are few, if any, more satisfactory ways of entering the medical profession at the present time than by joining a good college in Cambridge, passing the Previous, and then at once commencing the study of the sciences which form the groundwork of medicine, meanwhile devoting a certain moderate amount of time to attendance at the hospital, so as to obtain a general familiarity with the ways of sick folk and the methods of treatment. After the examinations in anatomy and physiology have been passed it is easy to adjourn to London, and return for the degree when the necessary clinical studies have been completed.

Addenbrooke's Hospital, at which the clinical instruction is given in Cambridge, contains only 158 beds, but it does a great deal of good work, and offers quite as large a field as is necessary for the first two or three years, during which the student's time is of necessity almost entirely engaged in working at anatomy, physiology, and the allied sciences.

The fee for pupilship at the Addenbrooke's Hospital is eight guineas.

### Owens College (Medical Department), Manchester.

This is one of the colleges of the Victoria University.

The Manchester Royal Infirmary, at which the medical instruction is given, contains 291 beds, and in connection with it there is a convalescent hospital at Cheadle (the Barnes Convalescent Hospital), containing 136 beds.

Instruction in mental diseases is given at the Royal Lunatic Asylum, and in infectious diseases at the Fever Hospital. Owens College, Manchester, of which the medical school is a department, is a large and most complete educational institution.

Large additions have lately been made to the buildings, so as to give the fullest possible opportunities for teaching in those departments more especially important to the students entering for medicine, namely, anatomy, physiology, pathology, and materia medica.

But in every direction Owens College is well equipped for its work.

The composition fee for the college courses is £70, and for the hospital practice 40 guineas.

University College (Medical Faculty), Liverpool.

This, again, is one of the colleges of the Victoria University. It possesses some very fine and well-equipped laboratories, some of which have only recently been opened, and more are still building, a new medical department being at the present time in course of erection. Many very liberal gifts have been made to this institution, and everything is being carried out in a most complete manner, so that it has become a thoroughly organised and well-equipped medical school.

Numerous scholarships and prizes, amounting to over £600, are awarded annually.

In connection with this college is the recently started and now well-known Liverpool School of Tropical Medicine.

The composition fee for the college classes is £70, and that for the hospital practice is 40 guineas.

The Yorkshire College (Medical Department), Leeds.

This also is one of the colleges of the Victoria University.

The Leeds Infirmary, at which the clinical instruction is given, has more than 400 beds in constant use, and has attached to it a convalescent hospital. Mental diseases are studied at the West Riding Asylum, and the practice of the Leeds Public Dispensary, the City Fever Hospital, and the Hospital for Women and Children is also open to the students. The new buildings for the Medical School were opened only four years ago, and they are very complete and well equipped.

The composition fee for the college courses is £67 4s., and that for the hospital practice 40 guineas.

University of Durham College of Medicine, Newcastle-upon-Tyne.

The Royal Infirmary, where the clinical instruction is given, contains 280 beds, but a large new hospital is being erected.

Residence at Newcastle for one year out of the five is necessary for all medical students desiring to graduate at the Durham University.

The composition fee for the college courses is 70 guineas, and that for the hospital practice 25 guineas, with small additional payments in certain cases.

The University of Birmingham (Faculty of Medicine).

To obtain a medical degree in the University of Birmingham it is necessary to have attended the classes of the University for at least the first four years of the curriculum.

The clinical instruction is given at the General Hospital and the Queen's Hospital, under the direction of the Clinical Board. These hospitals have a total of upwards of 400 beds, and are thoroughly well equipped for teaching purposes. The scientific teaching is given by the University professors and lecturers.

The composition fee for the lectures is 80 guineas, and that for the hospital practice 40 guineas.

University College (Faculty of Medicine), Bristol.

It is now arranged that students of the college shall be admitted to the clinical practice of the Bristol Royal Infirmary and the Bristol General Hospital conjointly. These institutions have between them a total of 470 beds, and complete departments for the various specialties.

The composition fee for the lectures is 65 guineas, and that for the hospital practice 35 guineas.

University College (Department of Medicine), Sheffield.

The hospitals at which the clinical instruction is given are the Sheffield Royal Infirmary, containing 240 beds, the Sheffield Royal Hospital with 125 beds.

The composition fee for the lectures is 60 guineas, and for hospital practice £45.

University College, Cardiff, School of Medicine.

Three out of the five years of the curriculum may be spent at this school, the courses of study being recognised as qualifying for the Preliminary Scientific and Intermediate Examination in Medicine in the University of London, and for the first and second examination under the Conjoint Board.

Clinical instruction is given at the Cardiff Infirmary.

### SCOTTISH MEDICAL SCHOOLS.

Edinburgh University.

School of Medicine of the Royal Colleges, Edinburgh.

Medical College for Women, Edinburgh.

Glasgow University.

Queen Margaret College, Glasgow (the Women's Department of the University).

St. Mungo's College, Glasgow.

Anderson's Medical College, Glasgow.

University of Aberdeen.

University College, Dundee. Connected with the University of St. Andrews.

### IRISH MEDICAL SCHOOLS.

The School of Physic in Ireland, Dublin.

The Catholic University Medical School, Dublin.

The Schools of Surgery of the Royal Colleges of Surgeons in Ireland, Dublin.

Queen's College, Belfast.

Queen's College, Cork.

